# Influence of the Seasons of the Year, Employment, etc., on the Gain or Loss of Weight by the Prisoners in the Convict Prison at Wakefield

**Published:** 1859-01

**Authors:** W. R. Milner

**Affiliations:** Surgeon.


### ON THE INFLUENCE OF THE SEASONS OF THE YEAR, EMPLOYMENT, PERIOD OF IMPRISONMENT, ETC., ON THE GAIN OR LOSS OF WEIGHT, BY THE PRISONERS CONFINED IN THE CONVICT PRISON AT WAKEFIELD, BETWEEN JANUARY 1, 1848, AND DECEMBER 31, 1857.

By W. R. MILNER, Surgeon.

THE prisoners upon whom my observations have been made, are men who have had sentences of transportation or penal servitude passed upon them, and who have been sent to Wakefield to undergo the first portion of their punishment, during which they are kept in separate cells for a period of about nine months.

The prisoners are all males, between the ages of sixteen and fifty, and are all presumed to be in good health when sent. The cells in which they are confined have a cubic capacity of about nine hundred feet, and from thirty to thirty-five cubic feet of air, per minute, are passed through each cell.

The mean temperature of the cells for the entire year was 61°; the highest monthly mean 66·5° occurred in August; the lowest 56·9° in March.

The diet is uniform, with the exception of the alterations ordered by the medical officer in individual cases, and consists of the following articles daily:—Bread, twenty ounces; meat without bone, four ounces; soup, half a pint (these are equivalent to about seven ounces and three quarters of butcher's meat); potatoes, one pound; skimmed milk, three quarters of a pint; gruel, one pint, containing two ounces of oatmeal.

The dress is, cloth jacket, waistcoat, trousers, cap, and stock; linen shirt; woollen stockings, drawers, and under shirt.

The prisoners are sent out to exercise in the open air nine hours a week; the exercise is for one hour at a time; the men walk in circles, and every ten minutes they run for a hundred and fifty yards. They are all supplied with work, and are for the greater part employed in making mats, and matting of cocoa fibre and other materials; some work at tailoring, and shoemaking, and a few have other work to do.

All the prisoners are weighed on admission, and at the latter end of every calendar month during their stay.

The number of prisoners over whom my observations extend has been four thousand; the period of time occupied, ten years; the average number weighed monthly, three hundred and seventy-two; and the total number of individual weighings, forty-four thousand and four.

The men have all been weighed by myself, or under my superintendence; and the series of observations has been unbroken.

The results of these weighings have been tabulated on various bases, with a view to isolate the effect of a certain number of variable conditions on the gain, or loss of weight, among these prisoners, and to determine the amount of influence exerted by each of these conditions.

The conditions which I have selected for investigation, are:

1. The season of the year;
2. The period of imprisonment;
3. The employment in prison;
4. The age of the prisoners on admission;
5. The height ditto.

The influence exerted by each of these conditions is well marked, and, with one exception, viz., the influence of season, the deductions are such as would have been anticipated. I am not, however, aware that any similar observations have yet been published extending over so long a period, applied to so large a number of men, and carried out under such favourable circumstances. I can speak confidently as to the care and accuracy employed, both in taking and in working up the observations; and therefore, although they may not bring out any new physiological laws, they may form useful raw material to be employed by some future labourer in the same field.

### INFLUENCE OF SEASON.

The first table shows the influence of the season of the year on the weight of a number of men placed during the entire year under circumstances of food, clothing, and work, which do not differ, and who for the greater part of the day are in a temperature which does not vary greatly between the hottest and the coldest months. Under such circumstances, it might be expected that the weight of the men, taken as a whole, would remain sensibly the same; and that the numbers losing or gaining, as well as the quantities lost or gained, would vary little month by month; or that, if any marked variation occurred, it would be of an accidental character, depending on the greater or less amount of sickness during any particular month, etc. The inspection of Table I, however, shows that a marked periodicity exists ; and that, taking an average of years, we have two distinct series of months, during the one of which there is a constant loss of weight, and during the other a constant gain ; so that, if we divide the year into quarters, there is a loss during the first and fourth quarters, and a gain during the second and third.

The two series of gaining and losing months are unbroken, except in one instance. On reference to the table it will be seen that in November, which is in the losing series, a gain occurred. The amount gained is very small ; and I believe the discrepancy was caused by the arrival of large numbers of prisoners in September and October. It will be shown under another head that the men usually gain weight for a short time after they are received, so that probably this break in the series results from the influence of the stage of imprisonment, rather more than balancing the influence of season. On looking down the columns which show the average gain or loss per prisoner weighed, it will be seen that, beginning at December, the amount lost per man increases rapidly, and very steadily, till March, but that between March and April there is a very abrupt transition from loss to gain. The gains then continue till August, the amount gained increasing on the whole, but by a series of jerks, each alternate month presenting a larger and a smaller gain respectively, so that to obtain a steadily increasing series we should have to couple the summer months in pairs. Between August and September a change of sign occurs, about equal in amount, but in the opposite direction to that which took place between March and April.

The changes between March-April, and August-September, are far greater in amount than the changes which take place between any other pairs of consecutive months ; and this remark applies with greater force to the percentages of men gaining or losing, and to the net gains and losses, than to the average gains or losses per man.

The inferences which I think may be fairly drawn from these observations, are :—

1. The body becomes heavier during the summer months, and the gain varies in an increasing ratio.

2. The body becomes lighter during the winter months, and the loss varies in an increasing ratio.

3. The changes from gain to loss, and the reverse, are abrupt and take place about the end of March, and the beginning of September.

The results which I have thus obtained from the discussion of a large number of periodical weighings, present a remarkable relation to the results obtained by Dr. Edward Smith, from a series of most valuable and elaborate experiments which he has made on the quantities of carbonic acid thrown off by the lungs at various seasons of the year.

For instance, Dr. Smith finds that the quantity of carbonic acid thrown off is much greater in winter than in summer. My weighings show that the prisoners lost weight in winter, when the evolution of carbonic acid was great, and gained weight in summer, when less carbonic acid was given out.

This in itself would be a striking coincidence; but it will be seen from Table I, that a sudden change took place between March and April, and at the same time of the year, Dr. Smith found that a similar change took place in the amount of carbonic acid thrown off, and that the *amount* of the change was much greater at that period than at any other time, and so much greater that the great alteration struck him as being a very remarkable circumstance. Dr. Smith's paper was read at the Leeds meeting of the British Association, and his observations did not extend to the August-September period. I am therefore, unable to say if any equally marked change takes place in autumn. There can be little doubt that variations of temperature, light, etc., are the principal agents in causing these changes, but I believe it will be found that in addition to the direct influence of these physical agents, a periodic action occurs in the system which adds to or diminishes the effect of the physical agents alone.

### STAGE OF IMPRISONMENT.

I have divided the time passed at Wakefield into periods of two months each, and have tabulated six of these periods, so as to show the variation of the weight of the men during the first twelve months of their stay. I have not carried the table any further, as very few prisoners remained longer than twelve months, and those who were kept beyond that time were chiefly invalids, and consequently, cases from which no general inferences could be fairly drawn.

The table shows the gains and losses in bi-monthly periods, and also the proportion of prisoners who had to be placed on the extra-diet list, who were put on the list during each period. The number placed on extra diet, during the first twelve months of their stay, was 1,393, out of which number 3·14 per cent. were put on during the first two months, and 12·31 per cent. during the second two months.

The stage of their imprisonment here had evidently a very marked effect; during the first two months the majority gained weight; in the second bi-monthly period a large loss occurred, equal to nearly twice the amount gained in the first period; in the third period there was still a loss, but not to so great an amount; the next three periods show a steadily increasing gain.

For a due understanding of these fluctuations, it is necessary to consider the circumstances under which prisoners are received into this prison. They are all brought from other prisons after having been tried, and sentenced to various periods of transportation, or penal servitude; they have consequently passed through the period of anxiety which elapses between committal and trial, during which time, I have reason to think, men often fall off very much in condition and health. When we receive them their fate is decided, and they know the worst. In a large proportion of cases, I believe this is followed by a feeling of relief, and by a reaction of the mind against the depression under which it had previously been suffering; later on, the continued imprisonment begins to tell, and it becomes necessary to give extra diet to counteract its depressing tendency. A reference to the tables shows that it was thought necessary to give extra diet to a large number of prisoners during the fifth, sixth, seventh, and eighth months.

The number of prisoners who were placed on the extra diet list for the first time during these four months was 833, being nearly twenty-one per cent. of the prisoners in confinement, and sixty per cent. of the whole number who were put on extra diet during the twelve months.

The effect of this addition to the diet is shown by the gradual and progressive improvement during the last three bi-monthly periods, when the amount gained, added to the gain of the first period, has nearly restored the equilibrium of the mass.

In the preceding tables, the basis of the calculations has been the monthly weighings; but in those which are to follow, the basis of the calculations is the difference between the weights of the prisoners when received, and when removed, and consequently represents the entire loss or gain during an average period of 326 days.

PRISON EMPLOYMENT.

In Table III, I have distributed the various employments of the prisoners into five groups, putting into each group the classes of workmen, who, as a class, were most nearly associated in the average amounts gained or lost during their stay, and, when arranged on this principle, it will be found that the groups also represent very accurately the amount of muscular force required to be expended in the respective kinds of work at which they were employed. The first group consists of men employed in picking coir, an occupation in which the labour is merely nominal; and it will be seen that these men gained nearly two pounds each on the average, and that a large per centage of them were gaining weight. The coir pickers are placed in a group by themselves, as they consist principally of exceptional cases, a large proportion of them being men who, from weakness or infirmity, were unfit for real labour; many were, on medical grounds, employed in the garden, and had extra allowances.

The next group contains men working at sedentary trades, as tailors, and shoemakers, as well as a few employed in writing, and other light occupations. Of these men a large per centage gained weight, and the average gain was nearly a pound and three quarters per man.

The group which follows comprises carpenters, mechanics, and men employed in winding the yarn into balls, or winding it on to bobbins for the mat-makers. The men in this group generally work standing, and therefore, a greater number of muscles have to be brought into play. The weight of work, however, is thrown on the arms, and the legs have little more to do than to support the body in a convenient attitude. A smaller per centage of these gained weight, and the average amount gained was less.

The fourth group contains the men employed in weaving canvas, in making mats in the loom, or on boards, and also a small number, thirty-six, who were engaged in plaiting coir, or in binding mats. The work of all these men is decidedly heavier than that of the men forming the preceding groups, and the majority of these were found to have lost weight.

The last group contains only one class of work, viz, the weaving of coir-matting; but the effects of this were so very decided, that it was necessary to give it a place to itself.

The weaving of coir-matting by hand is a very laborious occupation; the yarn is coarse and rough, so that the friction between the threads of the warp and weft is great, and to produce good firm work, the weft has to be heavily and repeatedly struck, in doing which the muscles of the arms and trunk are brought into powerful action; the legs have also to be employed in working the treddles, and, in consequence of the power required to work the loom, the weaver cannot work sitting.

The effect of this greater expenditure of muscular force is very manifest, for nearly eighty per cent. of the men so employed lost weight during their stay, and the average loss per man was nearly seven pounds.

The influence of the various employments would have been much more marked if it had not been, to some degree, counteracted by the extra diet given to those men who were falling off very much in weight, and the numbers to whom it was found necessary to give extra diet, in each class, also bore a pretty close relation to the amount of muscular force expended. Among the men employed in coir-picking, 26·8 per cent. had to be placed on extra diet; in the second group, 26·4 per cent.; in the third, 36·8 per cent.; in the fourth group, 39·4 per cent.; while of the matting weavers, 60·1 per cent. required additional food.

### AGE.

The average age of the four thousand prisoners when received, was twenty-six years and a half; above twenty-five per cent. were under twenty-one years old, and fifty-two per cent. were under twenty-four; a large proportion of them, therefore, were still growing.

When grouped according to age, as in Table IV, it appears that the younger men gained weight, the middle aged lost, and the older men gained; also, that the younger the men were, the greater the amouut gained. If, however, we were to infer from this, that imprisonment was less injurious to the young, or that the food supplied to them had been more than sufficient to keep them in health, we should be in error.

According to Quetelet, the average rate of increase for one year in males, is as follows:—

| | Age. | | Age. | | | lbs. |
|---|---|---|---|---|---|---|
| From | 17 | to | 18 | = | + | 8·5 |
| | 18 | „ | 19 | = | + | 4·4 |
| | 19 | „ | 20 | = | + | 3·9 |
| | 20 | „ | 25 | = | + | 1 5 |
| | 25 | „ | 30 | = | + | 0·3 |
| | 30 | „ | 40 | = | | 0 0 |
| | 40 | „ | 50 | = | — | 0·3 |

Now, the prisoners who were received at seventeen, ought, during their stay with us, averaging about 326 days, to have gained 7·59 pounds per man; but they only gained 2·81 pounds, consequently, there was an average virtual loss of 4·78 pounds; and, applying the same principle of correction to the other ages, it will be found that there was a virtual loss on all ages below forty in the following proportion:—

| Age. | lbs. |
|---|---|
| 17 | 4·78 |
| 18 | 1·61 |
| 19 | 2·42 |
| 20 | 1·39 |
| 21—24 | 1·33 |
| 25—30 | 0·37 |
| 30—40 | 0·90 |

The men above forty, gained 1·07 each.

If we take the prisoners at seventeen as one group, and then take the next six ages in pairs, we shall have the virtual losses represented by the following figures, at 17, 4·78 pounds; at 18-19, 2·02 pounds; at 20-24, 1·36 pound; at 25-40, 1·14 pound; showing a regular decrease in the virtual loss as the age increases.

The loss of weight is one of the data on which I form my opinion as to the necessity for giving a prisoner extra food, and I was not for some time so fully alive to the importance of giving a larger quantity of food to the young. The extra diets have therefore been distributed pretty equally over all ages; a rather larger proportion has fallen to the share of the younger prisoners, but the difference has been so slight as not to have been capable of producing any marked effect, and the figures may, I think, be taken to represent pretty fairly the relative wants of the system at different ages.

### HEIGHT.

The average height of the men, measured without their shoes, was 65·4 inches, and the number above and below that height were about equal.

By referring to Table V, it will be seen that the gains and losses in weight varied with the height, the shortest men gaining most; those between fifty-nine and sixty-two inches high, gaining less; between sixty-three and sixty-six, where the mean height falls, being stationary; those above this height losing, and the loss becoming gradually greater as the height increases.

These differences would have been much more marked, if the men had all been kept strictly to their ordinary diet; but it has always been found necessary to put a large proportion of the tall men on extra diet. Out of the total number who had more than the usual allowance of food, there were—Under fifty-nine inches, 20·0 per cent.; from fifty-nine to sixty-two, 25·9; sixty-three to sixty-six, 33·2; sixty-seven to seventy, 44·5; seventy to seventy-four, 56·9. Of the whole 4,000 prisoners, 1,414, or 35·3 per cent, were placed on extra diet.

The per centage of prisoners who were placed on extra diet does not, however, represent the whole case, for the length of time the prisoners were on extra diet, or, in other words, the time when it became necessary to give additional food, varied. Thus, of the prisoners who were placed on the extra diet list, those who were under sixty-three inches were on it, on the average, 3·99 months; sixty-three to sixty-six inches, 4·27 months; sixty-seven to seventy inches, 4·82 months; and the men of seventy-one to seventy-four inches, 5·65 months; so that the tall men, who had extra food allowed, had it nearly fifty per cent. longer than the short men.

From a consideration of the facts which I have collected, I think we may fairly infer that there is a periodic variation in the weight of man during the year, the six summer months being gaining, and the six winter months being losing months. The amounts gained or lost, gradually increase from the commencement till the termination of each period respectively; the change from the gaining to the losing period, and the converse, is, however, abrupt, and these changes take place at times not very distant from the vernal and autumnal equinoxes.

When men, differing in labour, age, and height, are fed on an uniform diet, the amount of which is not quite sufficient to keep the weight of the whole mass in equilibrium, one portion of them gains while the other loses weight.

(*a*). Those whose work requires a small expenditure of muscular force, gain weight; those whose work requires a large expenditure of muscular force, lose weight; and the loss of weight varies with the amount of muscular force expended.

(*b*). Those who are below twenty years of age, gain weight *actually*, but, as they do not gain as much as they ought to have done by growth in the time, they *virtually* lose; between twenty and forty, there is an actual loss; those above forty, gain, and the virtual losses of those below forty, vary inversely with the ages.

(*c*). Those below the average height, gain weight; those about the average height, remain stationary; while those above it, lose weight, and the amount of loss or gain increases, in proportion as the height varies from the mean. The amounts lost by the tall men are, however, rather larger than the amounts gained by the short men.

These facts show the impossibility of framing any uniform dietary which can be suitable to a body of men among whom great individual differences exist, and they also show how necessary it is to allow a large discretionary power of giving extra food, to a medical officer, having charge of men who are unable to obtain any food, except that supplied to them by the authorities under whom they are placed.

The results of this investigation show the fallacy of the absurd notion which some people have, that prisoners are pampered and fed up in prison; for there was an actual loss on the whole weighings, although 3,635 were under forty years of age. Now, according to Quetelet, the weight of man continues to increase up to forty years, and therefore, by natural growth, there should have been a gain upon those 3,635 men, if they had had food sufficient to make up for the wear of the system, and leave a margin for growth. Now, taking Quetelet's tables as representing the normal growth of man, and multiplying the annual increase as shewn in those tables by the number of men at each age, and by the average duration of their stay at Wakefield, the prisoners under forty, ought to have weighed 3,750 pounds more at departure, than on arrival, instead of which they weighed 1175·5 pounds less, and were. therefore, 4925·5 pounds lighter, when they went away, than they probably would have been if they had been at liberty, and could have had as much food as they had chosen to eat.

It is evident, therefore, that the prisoners have not been supplied with an excess of food.

In conclusion, I would observe that although the average net gains or losses is small in amount, the regularity with which they vary according to the variation of the conditions which have been made the subjects of comparison, proves the general correctness of the deductions drawn from them, especially when we consider how much the losses have been diminished by the quantities of extra diet which were given to those men in whom the ordinary diet was evidently not sufficient; and the tolerably regular manner in which the amounts of extra diet ran side by side with the losses, I think, proves that a proper care was exercised in selecting the cases in which additional food was thought necessary.

TABLE I.

| Month. | No. of prisoners weighed. | Number of Prisoners | | | Percentage of Prisoners | | | Number of Pounds | | Net | | Average | | | |
|---|---|---|---|---|---|---|---|---|---|---|---|---|---|---|---|
| | | Gaining weight. | Losing weight. | Station-ary. | Gaining weight. | Losing weight. | Station-ary. | Gained. | Lost. | Gain. | Loss. | Gain per prisoner gaining. | Loss per prisoner losing. | Gain per prisoner weighed | Loss per prisoner weighed |
| January - - | 3,879 | 1,645 | 1,918 | 316 | 42·4 | 49·4 | 8·2 | 3723·5 | 4274·0 | — | 550·5 | 2·26 | 2·25 | — | 0·14 |
| February - - | 3,747 | 1,561 | 1,808 | 378 | 41·6 | 48·3 | 10·1 | 3140·0 | 4051·0 | — | 911·0 | 2·01 | 2·24 | — | 0·24 |
| March - - - | 3,632 | 1,058 | 2,272 | 302 | 29·1 | 62·6 | 8·3 | 1918·5 | 5372·5 | — | 3454·0 | 1·81 | 2·37 | — | 0·95 |
| April - - - | 3,793 | 1,733 | 1,704 | 356 | 45·7 | 44·9 | 9·4 | 3710·0 | 3578·5 | 131·5 | — | 2·14 | 2·10 | 0·03 | — |
| May - - - | 3,673 | 1,627 | 1,694 | 352 | 44·3 | 46·1 | 9·6 | 3825·5 | 3795·0 | 30·5 | — | 2·35 | 2·24 | 0·01 | — |
| June - - - | 3,731 | 1,918 | 1,455 | 358 | 51·4 | 39·0 | 9·6 | 5322·0 | 3381·0 | 1941·0 | — | 2·77 | 2·32 | 0·52 | — |
| July - - - - | 3,511 | 1,622 | 1,546 | 343 | 46·2 | 44·0 | 9·8 | 3426·5 | 3135·5 | 291·0 | — | 2·11 | 2·04 | 0·08 | — |
| August - - - | 3,446 | 1,903 | 1,211 | 332 | 55·2 | 35·1 | 9·7 | 4879·5 | 2483·0 | 2396·5 | — | 2·56 | 2·05 | 0·70 | — |
| September - | 3,684 | 1,474 | 1,881 | 329 | 40·0 | 51·1 | 8·9 | 3303·0 | 4080·5 | — | 777·5 | 2·24 | 2·17 | — | 0·21 |
| October - - | 3,734 | 1,624 | 1,768 | 342 | 43·5 | 47·3 | 9·2 | 3515·0 | 3905·0 | — | 390·0 | 2·16 | 2·21 | — | 0·10 |
| November - - | 3,621 | 1,636 | 1,613 | 372 | 45·2 | 44·5 | 10·3 | 3345·5 | 3329·5 | 16·0 | — | 2·04 | 2·06 | 0·004 | — |
| December - - | 3,553 | 1,519 | 1,682 | 352 | 42·8 | 47·3 | 9·9 | 3237·0 | 3356·0 | — | 119·0 | 2·13 | 1·99 | — | 0·03 |
| First quarter - | 11,258 | 4,264 | 5,998 | 996 | 37·9 | 53·3 | 8·8 | 8782·0 | 13697·5 | — | 4915·5 | 2·06 | 2·29 | — | 0·44 |
| Second quarter | 11,197 | 5,278 | 4,853 | 1066 | 47·1 | 43·4 | 9·5 | 12857·5 | 10754·5 | 2103·0 | — | 2·44 | 2·22 | 0·19 | — |
| Third quarter | 10,641 | 4,999 | 4,638 | 1004 | 47·0 | 43·6 | 9·4 | 11609·0 | 9699·0 | 1910·0 | — | 2·32 | 2·09 | 0·17 | — |
| Fourth quarter | 10,908 | 4,779 | 5,063 | 1066 | 43·8 | 46·4 | 9·8 | 10097·5 | 10590·5 | — | 493·0 | 2·11 | 2·09 | — | 0·05 |
| Winter months | 22,166 | 9,043 | 11,061 | 2062 | 40·8 | 49·9 | 9·3 | 18879·5 | 24288·0 | — | 5408·5 | 2·09 | 2·90 | — | 0·24 |
| Summer mo. - | 21,838 | 10,277 | 9,491 | 2070 | 47·0 | 43·5 | 9·5 | 24466·5 | 20453·5 | 4013·0 | — | 2·38 | 2·16 | 0·18 | — |
| Whole year - | 44,004 | 19,320 | 20,552 | 4132 | 43·9 | 46·7 | 9·4 | 43346·0 | 44741·5 | — | 1395·5 | 2·24 | 2·18 | — | 0·03 |

### TABLE II.

| Stage of Imprisonment at Wakefield. | No. of prisoners weighed. | Number of Prisoners | | | Percentage of Prisoners | | | No. of Pounds | | Net | | Average | | | | Percentage placed on extra diet. |
|---|---|---|---|---|---|---|---|---|---|---|---|---|---|---|---|---|
| | | Gaining weight. | Losing weight. | Stationary. | Gaining weight. | Losing weight | Stationary. | Gained. | Lost. | Gain. | Loss. | Gain per prisoner gaining. | Loss per prisoner losing. | Gain per prisoner weighed | Loss per prisoner weighed | |
| First & second mo. | 7980 | 3901 | 3374 | 705 | 48·9 | 42·3 | 8·8 | 10028·0 | 8108·5 | 1929·5 | — | 2·57 | 2·40 | 0·24 | — | 3·14 |
| Third & fourth „ | 7880 | 2988 | 4141 | 751 | 37·9 | 52·6 | 9·5 | 5769·0 | 9383 0 | — | 3614·0 | 1·93 | 2·27 | — | 0·46 | 12·21 |
| Fifth & sixth „ | 7663 | 3090 | 3833 | 740 | 40·3 | 50·0 | 9·7 | 6562·5 | 8186·5 | — | 1624·0 | 2·12 | 2·14 | — | 0·21 | 29·81 |
| Seventh & eighth | 6715 | 2968 | 3044 | 703 | 44·2 | 45·3 | 10·5 | 6477·0 | 6009·0 | 468.0 | — | 2·18 | 1·97 | 0·07 | — | 29·98 |
| Ninth & tenth „ | 5211 | 2331 | 2319 | 561 | 44·7 | 44·5 | 10·8 | 5243·5 | 4630·0 | 613·5 | — | 2·25 | 2·0 | 0·12 | — | 15·98 |
| Eleventh & twelfth | 3277 | 1547 | 1363 | 367 | 47·2 | 41·6 | 11·2 | 3351·5 | 2800·5 | 551·0 | — | 3·17 | 2·05 | 6·17 | — | 8·88 |
| Total - - - | 38726 | 16825 | 18074 | 3827 | 43·4 | 46·7 | 9·9 | 37441·5 | 30117·5 | — | 1676·0 | 2·22 | 2·17 | — | 0·04 | 100·00 |

### TABLE III.

| Employment in Wakefield Prison. | No. of prisoners weighed. | Number of Prisoners | | | Percentage of Prisoners | | | No. of Pounds | | Net | | Average | | | | Percentage on extra diet. |
|---|---|---|---|---|---|---|---|---|---|---|---|---|---|---|---|---|
| | | Gaining weight. | Losing weight. | Stationary. | Gaining weight. | Losing weight. | Stationary. | Gained. | Lost. | Gain. | Loss. | Gain per prisoner gaining. | Loss per prisoner losing. | Gain per prisoner weighed | Loss per prisoner weighed | |
| Coir pickers - - | 71 | 40 | 23 | 8 | 56·3 | 32·4 | 11·3 | 311·5 | 181·5 | 130·0 | — | 7·79 | 7·89 | 1·83 | — | 26·8 |
| Tailors - - - / Shoemakers - / Miscellaneous - | 1411 | 812 | 524 | 75 | 57·6 | 37·1 | 5·3 | 5009·0 | 2601·5 | 5407·5 | — | 6·17 | 4·96 | 1·71 | — | 26·4 |
| Carpenters - - / Mechanics - - / Winders - - - | 269 | 147 | 107 | 15 | 54·6 | 39·8 | 5·6 | 966·0 | 686·0 | 324·0 | — | 6·56 | 5·94 | 1·20 | — | 36·8 |
| Mat makers - / Mat weavers - / Canvas weavers / Mat finishers - / Coir plaiters - | 2064 | 826 | 1152 | 86 | 40·0 | 55·8 | 4·2 | 4555·0 | 6881·5 | — | 2326·5 | 5·51 | 5·97 | — | 1·13 | 39·4 |
| Matting weavers - | 185 | 37 | 145 | 3 | 20·0 | 78·4 | 1·6 | 180·0 | 1441·0 | — | 1261·0 | 4·87 | 9·94 | — | 6·81 | 60·1 |
| Total - - - | 4000 | 1862 | 1951 | 187 | 46·5 | 48·8 | 4·7 | 11015·5 | 11740·5 | — | 725 | 5·92 | 6·02 | — | 0·18 | 35·3 |

TABLE IV.

| Age. | No. of prisoners weighed. | Number of Prisoners | | | Percentage of Prisoners | | | Number of Pounds | | Net | | Average | | | | Percentage on extra diet. |
|---|---|---|---|---|---|---|---|---|---|---|---|---|---|---|---|---|
| | | Gaining weight. | Losing weight. | Stationary. | Gaining weight. | Losing weight. | Stationary. | Gained. | Lost. | Gain. | Loss. | Gain per prisoner gaining | Loss per prisoner losing. | Gain per prisoner weighed | Loss per prisoner weighed | |
| 17 - - - - | 62 | 37 | 22 | 3 | 59·7 | 35·5 | 4·8 | 273·5 | 99·0 | 174·5 | — | 7·39 | 4·50 | 2·81 | — | |
| 18 - - - - | 288 | 180 | 100 | 8 | 62·5 | 34·7 | 2·8 | 1098·0 | 430·5 | 667·5 | — | 6·10 | 4·31 | 2·32 | — | |
| 19 - - - - | 334 | 178 | 136 | 20 | 53·3 | 40·7 | 6·0 | 989·0 | 632·5 | 356·5 | — | 5·56 | 4·65 | 1·07 | — | |
| 20 - - - - | 330 | 150 | 161 | 19 | 45·5 | 48·8 | 5·7 | 801·0 | 816·5 | — | 15·5 | 5·34 | 5·07 | — | 0·05 | |
| 21 — 24 - | 1059 | 444 | 577 | 38 | 41·9 | 54·5 | 3·6 | 2390·0 | 3518·5 | — | 1128·5 | 5·38 | 6·10 | — | 1·06 | |
| 25 — 30 - - | 940 | 438 | 460 | 42 | 46·6 | 49·0 | 4·4 | 2648·0 | 2999·5 | — | 351·5 | 6·05 | 6·52 | — | 0·37 | |
| 31 — 40 - - | 704 | 284 | 379 | 41 | 40·3 | 53·8 | 5·9 | 1830·5 | 2586·5 | — | 756·0 | 6·45 | 6·82 | — | 1·07 | |
| 41 & upwards | 283 | 151 | 116 | 16 | 53·3 | 41·0 | 5·7 | 985·5 | 657·5 | 328·0 | — | 6·53 | 5·67 | 1·16 | — | |
| Total - - | 4000 | 1862 | 1951 | 187 | 46·5 | 48·8 | 4·7 | 11015·5 | 11740·5 | — | 725·0 | 5·92 | 6·02 | — | 0·18 | |

TABLE V.

| Height in Inches. | No. of prisoners weighed. | Number of Prisoners | | | Percentage of Prisoners | | | Number of Pounds | | Net | | Average | | | | Percentage on extra diet. |
|---|---|---|---|---|---|---|---|---|---|---|---|---|---|---|---|---|
| | | Gaining weight. | Losing weight. | Stationary. | Gaining weight. | Losing weight. | Stationary. | Gained. | Lost. | Gain | Loss. | Gain per prisoner gaining. | Loss per prisoner losing. | Gain per prisoner weighed | Loss per prisoner weighed | |
| Under 59 - | 20 | 10 | 9 | 1 | 50·0 | 45·0 | 5·0 | 57·5 | 40·0 | 17·5 | — | 5·75 | 4·44 | 0·87 | — | 20·0 |
| 59 — 62 - | 567 | 278 | 267 | 22 | 49·1 | 47·1 | 3·8 | 1570·5 | 1332·5 | 238·0 | — | 5·65 | 4·99 | 0·42 | — | 25·9 |
| 63 — 66 - | 2343 | 1123 | 1103 | 117 | 47·9 | 47·1 | 5·0 | 6351·0 | 6308·0 | — | 43·0 | 5·66 | 5·72 | — | 0·02 | 33·2 |
| 67 — 70 - | 1005 | 422 | 537 | 46 | 42·0 | 53·4 | 4·6 | 2830·5 | 3782·5 | — | 952·0 | 6·71 | 7·04 | — | 0·95 | 44·5 |
| 71 — 74 - | 65 | 29 | 35 | 1 | 44·6 | 53·9 | 1·5 | 306·0 | 277·5 | — | 71·5 | 7·10 | 7·93 | — | 1·10 | 56·9 |
| Total - - | 4000 | 1862 | 1951 | 187 | 46·5 | 4·88 | 4·7 | 11015·5 | 11740·5 | — | 725·0 | 5·92 | 6·02 | — | 0·18 | 35·3 |

